# Prognostic significance of further axillary dissection in breast cancer patients with micrometastases & the number of micrometastases: a SEER population-based analysis

**DOI:** 10.4155/fsoa-2018-0008

**Published:** 2018-04-23

**Authors:** Liu Ying-Ying, Yu Tian-Jian, Liu Guang-Yu

**Affiliations:** 1 Department of Breast Surgery, Fudan University Shanghai Cancer Center, No. 270, Dongan Road, Xuhui District, Shanghai, 200032, PR China; 2 Department of Oncology, Shanghai Medical College, Fudan University, No. 130, Dongan Road, Xuhui District, Shanghai, 200032, PR China

**Keywords:** ALND, breast cancer, breast-conserving surgery, micrometastases, SLNB

## Abstract

**Aim:**

To investigate the benefits of axillary dissection in patients with micrometastases.

**Methods:**

A review of data from the Surveillance, Epidemiology, and End Results database was performed from 2004 to 2013. Kaplan–Meier curves, Cox regression models, and propensity score matching were utilized to comprehensively evaluate the cohort.

**Results:**

Multivariate analysis after propensity score matching showed that patients with one to two micrometastases did not substantially benefit from axillary lymph node dissection in breast cancer-specific survival (p = 0.725). However, a subgroup analysis indicated that axillary dissection may benefit estrogen receptor-negative patients. Moreover, patients who carried three micrometastases had a significantly lower crude hazard ratio in breast cancer-specific survival.

**Conclusion:**

Axillary lymph node dissection may have advantages in high-risk micrometastatic patients. Patients with three micrometastases should be treated with caution.

## Lay abstract

The current study demonstrated that among patients undergoing breast-conserving surgery following radiation with T1–T2 invasive breast cancer and one to two nodal micrometastases, there was no difference in the breast cancer-specific survival for patients with and without axillary lymph node dissection. Although negative results of the Z0011 and International Breast Cancer Study Group 23–01 trials have been reported, high-risk micrometastatic patients (e.g., estrogen receptor negative, young age) may be candidates for radical axillary treatment according to the unclear radiation field and higher recurrence rate. The preliminary assessment of  three micrometastatic patients showed a lower breast cancer-specific survival than patients with one to two micrometastases. This study provides a novel perspective to the American Joint Committee on Cancer with respect to micrometastases.

Sentinel lymph node biopsy (SLNB) has become the standard indication for patients who are clinically considered to be lymph node negative [1]. The American College of Surgeons Oncology Group Z0011 trial [2–4] recruited patients who were clinically node-negative but had limited metastatic sentinel lymph node (SLN) involvement (one to two metastatic SLNs) and underwent breast-conserving surgery (BCS), with randomization to one of two groups: one group that received SLNB alone versus a second group that underwent complete axillary lymph node dissection (ALND). Although this trial reached a conclusion that ALND may not be necessary for women with early sentinel node metastases, the feasibility of applying the results of Z0011 remained a matter of debate [5,6] with respect to several factors, including patient adherence, imbalance among groups, population selection bias, definition of the margin of the inferiority analysis and the follow-up rate. The strategy to tailor patients eligible for the Z0011 entry emerged as time progressed. Given that the International Breast Cancer Study Group (IBCSG) study included isolated tumor cells (ITCs) within their definition of micrometastatic foci, we cannot exclude the possibility that this definition concealed the true prognosis of N1mi patients.

According to recent guidelines, micrometastases are exclusively detected on histopathological examination and defined as deposits of tumor invasion in lymph nodes, which are greater than 0.2 mm and/or more than 200 cells but no greater than 2.0 mm. Micrometastases identified in the SLNs appear to affect the survival or recurrence of patients compared with nodal negative [7–9], whereas other studies have obtained the opposite results [10–12]. Although a comparison of pN1mi with pN0 or pN1 was beyond our focus, an increasing number of clinicians have omitted ALND in cases of pN1mi [13], partially because of its uncertain prognostic significance and the lack of belief that axillary dissection has further prognostic benefit. Moreover, a high frequency of the occurrence of lymphedema after ALND should be considered [14].

Although the American College of Surgeons Oncology Group Z0011 and IBCSG 23-01 trial results provided clinicians with a new treatment pattern in patients with 1–2 positive SLN metastases or micrometastases, this topic remains under debate, and there is no explicit indication for axillary clearance in the late guidelines. Two large database-based prospective studies have reported that compared with SLNB alone, SLNB with ALND completion does not appear to be associated with a substantially improved survival of patients with micrometastasis in the SLNs [15,16]. Notably, in Z0011, more than 80% of patients were estrogen receptor (ER) positive, approximately 70% had T1 primary tumors, and most importantly, approximately 40% had only micrometastases in the SLNs [4,6]. We minimized our focus on micrometastases to determine whether it was sufficient to perform SLNB alone in patients staged N1mi. Although IBCSG 23-01 has reported their results [17], 10% of patients who received mastectomy and micrometastases were not comprehensively analyzed.

Our study aimed to determine whether patients who underwent BCS and radiotherapy, with T1–T2, pN1mi invasive breast cancers and up to two positive regional lymph nodes, would benefit from complete ALND over a 10-year period. More importantly, we explored the presence of breast cancer patients in various subgroups (e.g., ER-negative or two micrometastases) that may support the administration of ALND. We also explored the difference in prognostic effects caused by three micrometastases, which comprised a rarely diagnosed group.

## Methods

### Data resource & patient selection

We used the Surveillance, Epidemiology, and End Results (SEER) database of the National Cancer Institute to identify breast cancer patients in this retrospective study. SEER*Stat version 8.3.2 was utilized to identify 579,986 female patients older than 18 years of age who had a breast cancer diagnosis from 2004 to 2013. We excluded patients identified by death certificate or autopsy and with incomplete survival data. We restricted this cohort based on the following inclusion criteria: histologically confirmed diagnosis, unilateral early breast cancer as the first primary tumor, positive or negative estrogen receptor status (ER), progesterone receptor status (PR), known grade and laterality, staged in T1–T2, pN1mi, M0, exact number of regional nodes examined and involved, and treatment via lumpectomy and at least beam irradiation after surgery. Information regarding the HER2 status and molecular subtypes was only available after 2010 and was thus beyond our analysis. Patients registered since 2014 were not included because we did not consider them to have reached a sufficient duration of follow-up. The SEER database also provides information regarding chemotherapy; however, the data are limited.

Specifically, patients who had T1–T2 tumors, were staged pN1mi, had no more than three positive lymph nodes, and who had experienced BCS and radiotherapy were our primary focus. The SEER database denoted that micrometastasis included cases with micrometastases no greater than 0.2 cm in size, identified by pN1mi. Although several micrometastastic foci may be identified in one SLN, we considered the number of lymph nodes involved as the number of micrometastases. In light of clinical practice and the limited research articles that describe the number of micrometastatic lymph nodes, women with no more than three micrometastases identified were considered ‘eligible’ patients, and additional revelations of micrometastases were regarded as a potential misclassification or informative error.

**Figure 1.**
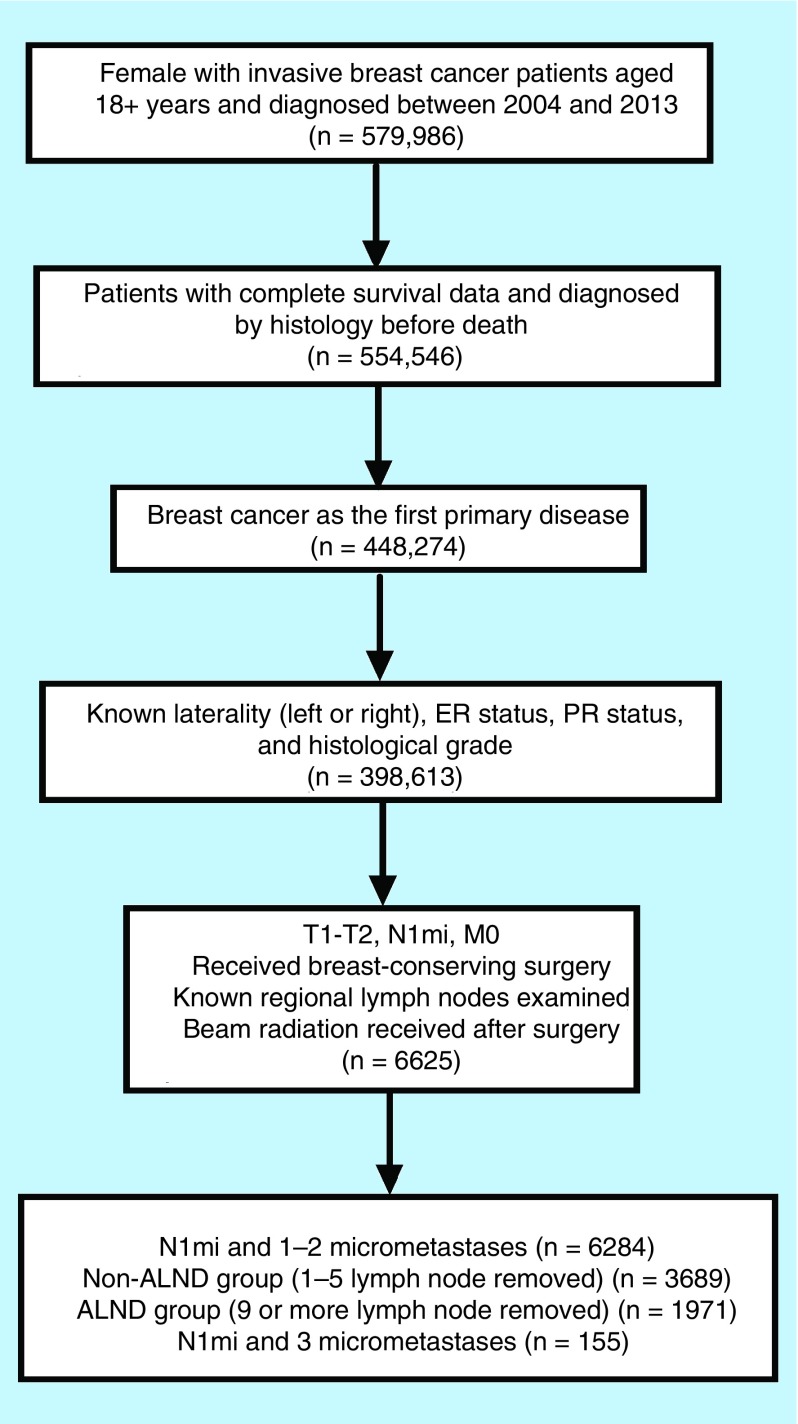
**Selection process of our defined population.** ALND: Axillary lymph node dissection; ER: Estrogen receptor; PR: Progesterone receptor status.

Patients were further categorized into two groups by the number of lymph node examinations (one to five categorized as non-ALND; nine or more categorized as ALND). The non-ALND group consisted of the patients undergoing SLNB alone (n = 3689), and the ALND group referred to the patients undergoing SLNB with complete ALND (n = 1971). This classification was consistent with two large register studies [15,16], which was better than roughly selecting four or five as a cut-off point [18–20]. Similarly, Schmocker RK *et al*. [21] suggested excluding a relatively small patient group (five, six, or seven lymph nodes examined) from analysis. Notably, the additional cases with three micrometastases were not included in our major analysis because of the limited number. Figure 1 presents the selection process and the final population included in our present analysis.

### Statistical analyses

We used SPSS 23.0 software to analyze the information obtained from the database. Descriptive statistics were performed to compare the baseline characteristics of the cohort according to their axillary treatment via Pearson's χ^2^ test. We stratified the age and year at diagnosis; ethnicity; tumor size and grade; ER, PR and HER2 status; positive number of lymph nodes involved and chemotherapy information. Propensity score matching was performed to verify an imbalance of patient characteristics. The SEER database does not provide information regarding local-regional recurrence. Therefore, the primary endpoint was breast cancer-specific survival. Survival curves were drawn via Kaplan–Meier analyses, and log rank tests were conducted with breast cancer-specific survival (BCSS) as the prognostic variable (Using GraphPad Prism 7.0) across different groups. The 5-year and 10-year BCSS rates were recorded.

The identification of factors significantly associated with BCSS was performed using the Cox regression model and the Wald test in SPSS 23.0. Univariate analyses and log–rank tests were conducted. Multivariate analyses were performed using a Cox regression model, and the factors taken into consideration are presented at the bottom of each table. Moreover, propensity score matching was utilized to overcome a potential selection bias and identify significant predictors of prognosis. The baseline characteristics were matched well; however, 268 patients in the ALND group were excluded during the matching process. Subgroup analyses were also performed to determine whether further ALND could benefit different groups. Hazard ratios (HRs) with 95% CIs were calculated as estimated risks of death. p-values were derived from two-tailed tests, and p < 0.05 was considered statistically significant.

## Results

### Descriptive statistics

**Figure 2.**
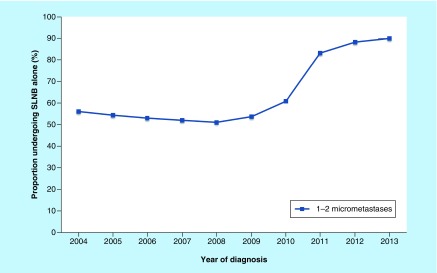
**Tendency of patients with one to two micrometastases undergoing sentinel lymph node biopsy alone between 2004 and 2013.** SLNB: Sentinel lymph node biopsy.

A total of 5660 patients with up to two SLN micrometastases treated with BCD and radiotherapy were available for the axillary treatment analysis from 2004 to 2013. After applying self-defined criteria, 3689 patients were included in the non-ALND group and 1,971 patients were included in the ALND group. Of the population in this cohort, the number of patients who received SLNB alone from 2004 to 2013 increased, with a substantial increase from the year 2011 (Figure 2). Our research using the SEER database suggested an increasing trend toward omitting ALND when SLNB indicated one to two micrometastases.

Of this entire cohort, 65.2% underwent SLNB alone. The median number of SLNs identified was two (range of one to five), and the median number of nodes during axillary dissection was 14 (range 9–48). In 88.6% of the cases, only one positive SLN was harvested. The patient and tumor baseline characteristics are presented in Table 1. There were a series of significant differences between the groups. The elder group or patients diagnosed in recent years were more likely to omit ALND. Compared with the other two subgroups, blacks were far more likely to be treated with SLNB alone. Regarding clinicopathologic factors, patients were more likely to undergo SLNB alone if they had a smaller tumor size or lower grade, a positive ER or PR status, or a negative HER2 status. Different molecular subtypes also influenced the decision regarding axillary treatment. The most aggressive molecular subtype, triple-negative breast cancers, exhibited a double proportion in the ALND group. In terms of adjuvant chemotherapy, 70% of the patients in the ALND group received treatment, a higher percentage than the non-ALND group, as previous studies have demonstrated [22,23]. Our study particularly focused on the number of positive lymph nodes with micrometastases, and we identified a substantial increase in the decision of axillary dissection from 8 to 18% when two positive lymph nodes were identified (p < 0.0001).

**Table 1.** **Baseline characteristics between nonaxillary lymph node dissection group and axillary lymph node dissection group.**

**Groups**	**Subgroups**	**Non-ALND (n = 3689)**		**ALND (n = 1971)**		**Total (n = 5660)**		**p-value**

		**No.**	**%**	**No.**	**%**	**No.**	**%**	
Age								<0.001

	18–50 years	893	24	633	55	1526	27	

	51–70 years	2032	55	1082	13	3114	55	

	>70 years	764	21	256	32	1020	18	

Race								<0.001

	White	3080	84	1561	79	4641	82	

	Black	296	8	248	13	544	10	

	Others/unknown	313	8	162	8	475	8	

Year of diagnosis								<0.001

	2004–2008	1418	38	1241	63	2659	47	

	2009–2013	2271	62	730	37	3001	53	

Tumor grade								<0.001

	I	952	26	406	21	1358	24	

	II	1825	49	896	34	2721	48	

	III/IV	912	25	849	45	1581	28	

Tumor size								0.009

	≤2 cm	2617	71	1334	68	3951	70	

	>2 cm, ≤3 cm	819	22	463	23	1282	23	

	>3 cm, ≤5 cm	253	7	174	9	427	7	

ER status								<0.001

	Positive	3367	91	1671	85	5038	89	

	Negative	322	9	300	15	622	11	

PR status								<0.001

	Positive	3055	83	1497	76	4552	80	

	Negative	634	17	474	24	1108	20	

HER2 status (n = 2400)								0.004

	Positive	153	12	56	8	209	9	

	Negative	1786	88	405	92	2191	91	

Molecular subtype (n = 2400)								<0.001

	HR^+^/HER2-	1686	87	361	78	2047	85	

	HR^+^/HER2^+^	129	7	48	10	177	8	

	HR^-^/HER2^+^	24	1	8	2	32	1	

	Triple negative	100	5	44	10	144	6	

Positive SLNs								<0.001

	1	3406	92	1607	82	5013	89	

	2	283	8	364	18	729	11	

Chemotherapy								<0.001

	Yes	1815	49	1372	70	3187	56	

	No/unknown	1874	51	599	30	2473	44	

Median number of LN removed		2 (1-5)		14 (9-48)				

ALND: Axillary lymph node dissection; ER: Estrogen receptor; HER2: Human epidermal receptor 2; LN: Lymph node; PR: Progesterone receptor; SLN: Sentinel lymph node.

### Survival analyses in whole cohort

**Figure 3.**
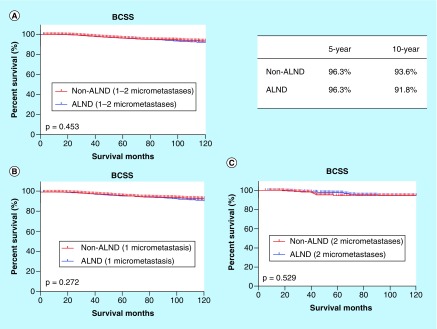
**Survival analyses of patients with 1–2 micrometastases according to axillary treatment.** ALND: Axillary lymph node dissection; BCSS: Breast cancer-specific survival.

Survival curves according to the axillary treatment are shown in Figure 3. There were no differences in the BCSS between the non-ALND and ALND groups (p = 0.453) (Figure 3A). The BCSS rates at 5 and 10 years were respectively 96.3 and 96.3% in the patients without ALND and 93.6 and 91.8% in the patients with ALND. Although a high percentage of patients had only one lymph node examined and concurrently showed positive results, the patients stratified by axillary treatment showed no significant difference in the BCSS (p = 0.272) (Figure 3B). The counterpart comparison was performed in the patients with two micrometastases, and no significant differences were identified (p = 0.529) (Figure 3C).

We evaluated a series of potential prognostic factors and determined their effects on the BCSS (Table 2, left). Our univariate analysis showed that older age, black race, a larger tumor size and higher grade and the absence of ER and PR expression were significantly associated with a shortened BCSS. After adjustment for confounding factors, the multivariate analysis indicated that the impacts of age, race, tumor size and grade, and ER and PR expression were maintained. A trend toward an improved BCSS was identified with chemotherapy administration (p = 0.089). Whether patients underwent ALND did not affect the BCSS (p = 0.783). Moreover, a breast cancer-specific survival difference according to the number of micrometastases was not identified (p = 0.477).

**Table 2.** **Univariate and multivariate analyses of breast cancer-specific survival in unmatched and matched cohort.**

**Groups**	**Subgroups**	**Unmatched cohort**			**Matched cohort**		

		**Breast cancer-specific survival**			**Breast cancer-specific survival**		

		**Log-rank p-value**	**Cox model p-value**	**HRs^†^ (95% CI)**	**Log-rank p-value**	**Cox model p-value**	**HRs^†^ (95% CI)**
Age		<0.001			<0.001		

	18–50 years		–	1.00			1.00

	51–70 years		0.869	0.97 (0.69–1.37)		0.603	0.90 (0.60–1.35)

	>70 years		<0.0001	2.72 (1.82–4.08)		<0.001	2.94 (1.76–4.91)

Race		<0.001			0.001		

	White		–	1.00			1.00

	Black		0.002	1.73 (1.21–2.47)		0.006	1.83 (1.19–2.83)

	Others/unknown		0.947	0.98 (0.58–1.67)		0.664	0.83 (0.36–1.91)

Tumor size		<0.001			<0.001		

	≤2 cm		–	1.00			1.00

	>2 cm, ≤3 cm		<0.0001	2.17 (1.60–2.96)		<0.001	2.33 (1.62–2.36)

	>3 cm, ≤5 cm		<0.0001	3.37 (2.31–4.93)		<0.001	3.55 (2.20–5.72)

Tumor grade		<0.001			<0.001		

	I		–	1.00			1.00

	II		0.003	2.57 (1.39–4.75)		0.008	3.15 (1.35–7.39)

	III/IV		<0.0001	4.49 (2.38–8.46)		0.001	4.55 (1.90–10.95)

ER status		<0.001			<0.001		

	Negative		–	1.00			1.00

	Positive		0.009	0.58 (0.38–0.87)		0.021	0.55 (0.33–0.91)

PR status		<0.001			<0.001		

	Negative		–	1.00			1.00

	Positive		<0.0001	0.47 (0.32–0.68)		<0.001	0.42 (0.26–0.68)

Axillary treatment		0.460			0.460		

	Non-ALND		–	1.00			1.00

	ALND		0.783	0.96 (0.73–1.27)		0.725	0.94 (0.68–1.31)

Positive SLNs		0.409			0.409		

	1		–	1.00			1.00

	2		0.477	0.86 (0.56–1.32)		0.838	0.94 (0.53–1.67)

Chemotherapy		0.383			0.383		

	No/unknown		–	1.00			1.00

	Yes		0.089	0.74 (0.52–1.05)		0.041	0.63 (0.40–0.98)

^†^Adjustment for race, age, tumor size and tumor grade.

ER status, PR status, axillary treatment, positive number of SLNs, adjuvant chemotherapy.

ALND: Axillary lymph node dissection; ER: Estrogen receptor; HR: Hazard ratio; PR: Progesterone receptor; SLN: Sentinel lymph node.

All imbalances between two groups were corrected through propensity score matching (Table 3). In our matched cohort, the univariate analysis of breast cancer-specific survival showed similar results to that of prior matching (Table 2, right). The subsequent multivariate analysis also indicated that old age (>70 years), a large tumor size (>2 cm, ≤5 cm), high grade and ER and PR negative remained poor prognosticators for BCSS. Furthermore, chemotherapy administration was associated with a prolonged BCSS (p = 0.041). As expected, the patients with and without ALND were not significantly different regarding the BCSS (p = 0.725). Moreover, one or two micrometastases carried the same prognostic significance (p = 0.838).

**Table 3.** **Patient demographics after propensity score matching. **

**Groups**	**Subgroups**	**Non-ALND (n = 1703)**		**ALND (n = 1703)**		**Total (n = 3406)**		**p-value**

		**No.**	**%**	**No.**	**%**	**No.**	**%**	
Age								0.881

	18–50 years	502	30	515	30	1017	30	

	51–70 years	969	57	956	56	1925	57	

	>70 years	232	14	232	14	464	14	

Race								0.970

	White	1412	83	1417	83	2829	83	

	Black	180	11	178	11	358	11	

	Others/unknown	111	7	108	6	219	6	

Tumor size								0.322

	≤2 cm	1161	68	1197	70	2358	69	

	>2 cm, ≤3 cm	418	25	381	22	799	24	

	>3 cm, ≤5 cm	124	7	125	7	249	7	

Tumor grade								0.951

	I	360	21	362	21	722	21	

	II	810	48	801	47	1611	47	

	III/IV	533	31	540	32	1073	32	

ER status								0.639

	Negative	205	12	214	13	419	12	

	Positive	1498	88	1489	87	2987	88	

PR status								0.214

	Negative	389	23	359	21	748	22	

	Positive	1314	77	1344	79	2658	78	

Positive SLNs								0.957

	1	1509	89	1510	89	3019	89	

	2	194	11	193	11	387	11	

Chemotherapy								0.971

	Yes	560	33	561	33	1121	33	

	No/unknown	1143	67	1142	67	2285	67	

Median number of LNs removed		2 (1–5)		14 (9–43)				

A total of 268 patients derived from ALND group were excluded during propensity score matching.

ALND: Axillary lymph node dissection; ER: Estrogen receptor; LN: Lymph nodel; PR: Progesterone receptor; SLN: Sentinel lymph node.

**Figure 4.**
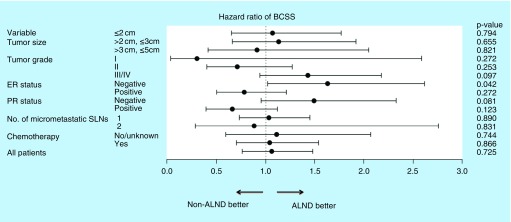
**Subgroup analyses assessing the benefit of further axillary lymph node dissection according to certain characteristics.** ALND: Axillary lymph node dissection; BCSS: Breast cancer-specific survival; ER: Estrogen receptor; PR: Progesterone receptor status.

To identify the specific subgroups that may benefit from further axillary dissection, subgroup analyses were performed in the matched cohort (Figure 4). Notably, a significantly shorter BCSS was identified when ALND was forgone in patients without ER expression (p = 0.042). The stratified survival analysis also showed a close to statistically significant improvement for the ALND group in BCSS (p = 0.081) among PR-negative patients.

### Prognostic difference caused by the number of micrometastases

**Figure 5.**
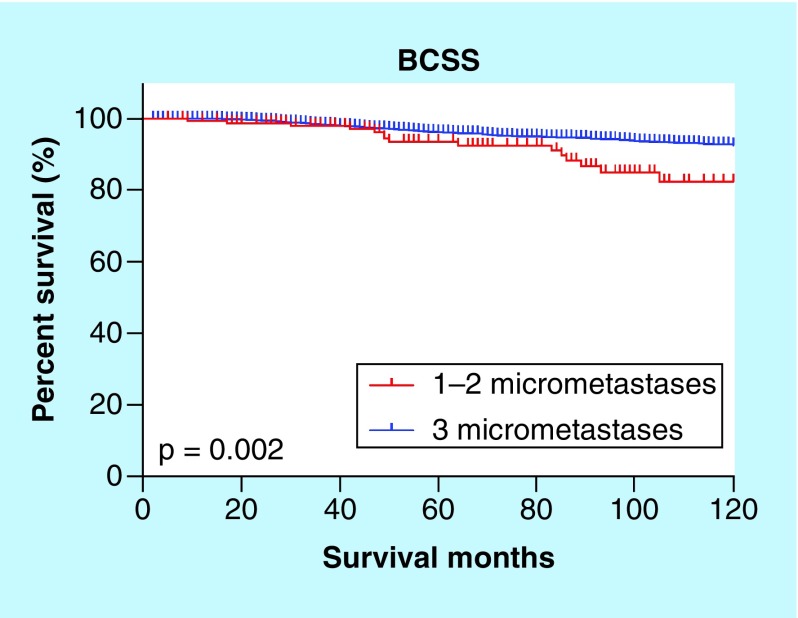
**Breast cancer-specific survival curve for the presence of one to two and three micrometastases.** BCSS: Breast cancer-specific survival.

The Kaplan–Meier curve suggested that three micrometastases appeared to be a controversial diagnosis because it tended to be associated with worse survival (p = 0.002) (Figure 5). An adjuvant therapy indication is based on the comprehensive consideration of poor prognostic features; however, the SEER database does not provide endocrine therapy data and all patients in our cohort received radiotherapy. An additional multivariate analysis to adjust for chemotherapy was performed. Similarly, the analysis adjusted for chemotherapy and the multivariate analysis indicated that the patients with three micrometastases had a significantly worse BCSS than the patients with fewer micrometastases (Table 4).

**Table 4.** **Cox regression model for breast cancer-specific survival in patients with one to three micrometastases.**

**Number of micrometastases**	**HR (95% CI)^†^**	**p-value**	**HR (95% CI)^‡^**	**p-value**
1–2 micrometastases (n = 6284)	1.00	0.003	1.00	0.006

3 micrometastases (n = 155)	2.22 (1.31–3.75)	–	2.10 (1.24–3.56)	–

^†^Adjustment for administration of chemotherapy.

^‡^Adjustment for race, age, tumor size, tumor grade, estrogen receptor status, progesterone receptor status, positive number of sentinel lymph nodes and adjuvant chemotherapy.

BCSS: Breast cancer-specific survival; HR: Hazard ratio

## Discussion

Yi *et al*. [16] reported that the proportion of patients with microscopic SLN disease who did not undergo complete ALND substantially increased from 1998 to 2004. We determined that the treatment of the axilla changed between 2004 and 2013, with a sharp increase since 2011. The Z0011 and IBCSG 23-01 results may have attracted substantial attention among clinicians and provide new hope to many women who expect to avoid the potential side effects of ALND.

Previous studies have attempted to address the question of whether axillary clearance may be safely omitted when micrometastases are identified in SLNs. It is possible that not performing ALND in SLN positive patients is only safe when patients underwent BCS because subsequent radiotherapy may benefit them by treating an uncertain axillary lymph node status. Furthermore, although micrometastases are relatively rare and it is difficult to obtain a large study population, the SEER database provides a tremendous population to retrospectively explore this pending question. In our cohort, we restrained our patients by BCS and radiotherapy offered. The micrometastatic status was confined to no more than two identified micrometastases because previous studies have reported nearly all patients had only one or two positive results. We determined that ALND may be safely omitted in our defined patients, which is consistent with previous studies of patients with micrometastases [15–17,22–28], including two randomized trials. However, Youssef *et al*. [29] identified a significant difference in overall survival; Houvenaeghel *et al*. [30] suggested that patients with micrometastases had increased recurrence rates and a shorter OS when ALND was omitted. In our matched population, race, age, malignancy grade, tumor size, hormone receptor (HR) status and chemotherapy use, but not the axillary treatment or number of micrometastases, were significantly associated with prognosis in the multivariate analysis. For individuals, these significant factors must be considered to provide the best treatment strategy [31]. Interestingly, patients who were ER-negative may benefit from further ALND, considering the subgroup analysis results. A recent questionnaire showed a swing status in radiation field design among high-risk micrometastatic patients, which may indirectly prompt the significance of radical axillary treatment [32]. However, there is insufficient evidence in high-risk patients. Strangely, both the Z0011 and IBCSG 23-01 trials have more events among ALND groups [4,17], which may explain the lack of a survival difference among ER-negative patients [4]. In particular, it nearly reached the cut-off value among the ER- and PR-negative group [17]. One prospective study aimed to apply the findings of the Z0011 trial among 242 younger patients or patients with triple-negative or HER2 overexpressing (HER2^+^) breast cancers [33]. Although there was no difference in axillary tumor burdens between the groups, the recurrence rate in high-risk cases was higher than that in average-risk patients (2.1 vs 0.4%, respectively) [33].

Notably, numerous studies have emphasized the importance of adjuvant systematic therapy among patients with micrometastases [25,29,31,34,35]. We determined that patients were more likely to receive chemotherapy in the ALND group. A potential reason for this phenomenon is that the patients submitted to SLNB alone frequently had a lower tumor burden or they did not consider radical therapy to be a better method. Our results were for reference only because this database did not provide hormone therapy information, and no survival analysis was completed due to incomplete chemotherapy and patient status information.

The major limitation of this study is that the SEER database does not specifically describe whether patients were treated with SLNB before complete ALND. Bilimoria *et al*. [15] analyzed patients with microscopic nodal metastases in the National Cancer Data Base from 1998 to 2005. In their research, patients were considered to have undergone SLNB with ALND completion if they had five or fewer nodes examined, whereas if they had at least nine nodes examined, the patients were deemed to have undergone an SLNB with ALND completion. The method proved to be relatively reasonable [15]. Yi *et al*. [16] reviewed 26,986 patients with positive SLNs (macrometastatic or micrometastatic) in the SEER database during 1998–2004, and the method to define the two groups was as same as Bilimoria's method. The advantage of this study is that we focused on micrometastases that were commonly harvested in SLNs.

It is noted that several studies have reported the specific situation of SLN examination and the number of micrometastases, and some studies included ITC in their analysis. In this study, 54.1% of the patients had one to two SLNs removed, which was similar to previous research [26,30,36]. Moreover, the median and range of SLNs in our study were also consistent with previous research [17,24,26,27,29,30,36]. However, two studies showed both a higher median number and wider range [37,38]. Regarding the micrometastatic SLNs, most studies have indicated an extremely high percentage of one and two positive SLNs [17,24,30,39], and several studies selected one as a cut-off point [36,37,40] without demonstrating additional details concerning the low quantity; other studies have shown no more than two invaded [25,27,29]. The relative small probability to discover three micrometastases in SLNs was caused by the low number of SLN examinations. Regarding the IBCSG 23-01 randomized controlled trial, one or two sentinel nodes were removed in 82% of patients [17], and only one patient was reported to have three micrometastases.

Furthermore, more than 80% of patients were commonly diagnosed with one micrometastasis, and few studies have described the diagnosis of three micrometastases; the IBCSG 23-01 trial reported one patient with three micrometastases. Therefore, we focused on the prognostic effects using the exact number of positive lymph nodes. We showed that there was no difference in the BCSS between one and two micrometastatic SLNs, regardless of the adjustment for confounding factors. To identify the probable difference in the number of micrometastases, there was a certain number of N1mi patients with four or more micrometastases, and of the nine examined lymph nodes, nine were positive. Notably, the American Joint Committee on Cancer (AJCC) defined ‘Micrometastases’, but not ‘Micrometastasis’, as positive lymph nodes greater than 0.2 mm but no greater than 2.0 mm without stating the quantity problem. Therefore, based on our clinical practice, we believed these cases were misclassified and incorrectly recorded or diagnosed; thus, we removed them from our analysis. From this point, it was illustrated by our preliminary analysis result that patients with three micrometastases had substantially worse breast cancer-specific survival (Figure 5 & Table 4). Although the SEER database included a large population from approximately 30% of the population in the USA, the finite cases of this subgroup limited division into two axillary treatment models. Here, we emphasized that N1mi stage patients diagnosed with three micrometastases or more were not so-called ‘Qualified Micrometastases’.

Other limitations of this study should be recognized. Firstly, this study is limited by its retrospective nature; there is an inevitable selection bias compared with randomized controlled clinical trials. However, we attempted to obtain an unbiased comparison using propensity score matching. Moreover, if further ALNDs are administered in patients with one to two micrometastatic SLNs, there is a low likelihood (7.1–18%) of identifying one or more non-SLN metastases [4,17,23,25–27,29,30,36,38,40], with several studies combining micrometastases and ITCs as a category. Thus, we could not consider these missing patients, who originally should have been in our ALND group. Furthermore, patients with an inadequate ALND (with five lymph nodes) may have been wrongly considered as patients in the group with SLN only; however, micrometastases included a description for the sentinel node in most cases. Finally, the SEER database does not provide detailed data regarding recurrence and the use of radiotherapy, which may have reduced the credibility of our results. We employed cases of breast-conserving therapy administered in the current study to avoid bias in the field of radiation and believed that most patients would have undergone standard whole breast irradiation.

## Conclusion

The current findings demonstrated that among patients undergoing BCS following radiation with T1–T2 invasive breast cancer and one to two nodal micrometastases, there was no difference in the BCSS for patients with and without ALND completion. This study provided additional support for the growth trend of omitting ALND when considering these cases as a whole. The subgroup analysis suggested that the absence of ER expression combined with other high-risks may be an indication of further ALND. Individualized axillary management strategies based on certain risk factors should be further investigated. This study also preliminarily clarified that three micrometastases may cause a worse BCSS in contrast to one to two micrometastases. Moreover, we suggest that the number of ALNs with micrometastases should be reported in the final report because of its prognostic significance, and a proposal of changes may be provided to the AJCC for additional future studies. The need to be careful during diagnosis and comprehensively evaluate the involved lymph nodes is highlighted.

## Future perspective

A longer follow-up in the Z0011 and IBCSG 23-01 trials will provide a more convincing result. However, the necessity of ALND in patients with multiple high-risk factors should be reported separately and investigated. A better radiation strategy should also be established.

**Summary points**

- Sentinel lymph node biopsy (SLNB) has become the standard indication for patients who are clinically considered to be lymph node negative.
- Micrometastases identified in the sentinel lymph nodes appear to affect the survival or recurrence of patients compared with nodal negative, whereas other studies obtained the opposite results.
- The current study aimed to explore whether patients who underwent breast-conserving surgery and radiotherapy, with T1–T2, pN1mi-invasive breast cancers, and one to two positive regional lymph nodes, would benefit from complete axillary lymph node dissection (ALND).
- We demonstrated that among patients undergoing breast-conserving surgery, following radiation with T1–T2 invasive breast cancer, and one to two nodal micrometastases, there was no difference in the breast cancer-specific survival for patients with and without ALND completion.
- These results provide additional support to the growth trend of omitting ALND in patients with one to two micrometastases in sentinel lymph nodes.
- Individualized axillary management strategies based on certain risk factors should be further investigated.
- We also preliminarily clarified that three micrometastases may cause a worse breast cancer-specific survival in contrast to one to two micrometastases.
- We expect additional research regarding the number of axillary lymph nodes with micrometastases as a result of its prognostic significance, and the proposal of new changes may be warranted to the American Joint Committee on Cancer with additional future studies.

## References

[1] Lyman GH, Giuliano AE, Somerfield MR (2005). American Society of Clinical Oncology Guideline recommendations for sentinel lymph node biopsy in early-stage breast cancer.

[2] Giuliano AE, Mccall L, Beitsch P (2010). Locoregional recurrence after sentinel lymph node dissection with or without axillary dissection in patients with sentinel lymph node metastases: the American College of Surgeons Oncology Group Z0011 randomized trial.

[3] Giuliano AE, Hunt KK, Ballman KV (2011). Axillary dissection vs no axillary dissection in women with invasive breast cancer and sentinel node metastasis: a randomized clinical trial.

[4] Giuliano AE, Ballman KV, Mccall L (2017). Effect of axillary dissection vs no axillary dissection on 10-year overall survival among women with invasive breast cancer and sentinel node metastasis: the ACOSOG Z0011 (Alliance) randomized clinical trial.

[5] Latosinsky S, Berrang TS, Cutter CS (2012). CAGS and ACS Evidence Based Reviews in Surgery. 40. Axillary dissection versus no axillary dissection in women with invasive breast cancer and sentinel node metastasis.

[6] Caudle AS, Hunt KK, Kuerer HM (2011). Multidisciplinary considerations in the implementation of the findings from the American College of Surgeons Oncology Group (ACOSOG) Z0011 study: a practice-changing trial.

[7] Andersson Y, Frisell J, Sylvan M, De Boniface J, Bergkvist L (2010). Breast cancer survival in relation to the metastatic tumor burden in axillary lymph nodes.

[8] Boer MD, Deurzen CV, Dijck JV (2009). Micrometastases and isolated tumor cells: relevant and robust or rubbish? (MIRROR): preliminary results of the MIRROR study from the Dutch breast cancer trialists’ group (BOOG).

[9] Chen SL, Hoehne FM, Giuliano AE (2007). The prognostic significance of micrometastases in breast cancer: a SEER population-based analysis.

[10] Gobardhan PD, Elias SG, Madsen EV (2011). Prognostic value of lymph node micrometastases in breast cancer: a multicenter cohort study.

[11] Ozao-Choy J, Giuliano AE (2011). Prognostic significance of micrometastasis and isolated tumor cells in the sentinel lymph node.

[12] Houvenaeghel G, Classe JM, Garbay JR (2014). Prognostic value of isolated tumor cells and micrometastases of lymph nodes in early-stage breast cancer: a French sentinel node multicenter cohort study.

[13] Wasif N, Ye X, Giuliano AE (2009). Survey of ASCO members on management of sentinel node micrometastases in breast cancer: variation in treatment recommendations according to specialty.

[14] Langer I, Guller U, Berclaz G (2007). Morbidity of sentinel lymph node biopsy (SLN) alone versus SLN and completion axillary lymph node dissection after breast cancer surgery: a prospective Swiss multicenter study on 659 patients.

[15] Bilimoria KY, Bentrem DJ, Hansen NM (2009). Comparison of sentinel lymph node biopsy alone and completion axillary lymph node dissection for node-positive breast cancer.

[16] Yi M, Giordano SH, Meric-Bernstam F (2010). Trends in and outcomes from sentinel lymph node biopsy (SLNB) alone vs. SLNB with axillary lymph node dissection for node-positive breast cancer patients: experience from the SEER database.

[17] Galimberti V, Cole BF, Zurrida S (2013). IBCSG 23-01 randomised controlled trial comparing axillary dissection versus no axillary dissection in patients with sentinel node micrometastases.

[18] Wang J, Mittendorf EA, Sahin AA (2014). Outcomes of sentinel lymph node dissection alone vs. axillary lymph node dissection in early stage invasive lobular carcinoma: a retrospective study of the surveillance, epidemiology and end results (SEER) database.

[19] Wong SM, Freedman RA, Stamell E (2015). Modern trends in the surgical management of Paget's disease.

[20] Wu Q, Li J, Sun S (2016). Breast carcinoma *in situ*: an observational study of tumor subtype, treatment and outcomes.

[21] Schmocker RK, Caretta-Weyer H, Weiss JM (2014). Determining breast cancer axillary surgery within the surveillance epidemiology and end results – Medicare database.

[22] Tvedskov TF, Jensen MB, Ejlertsen B, Christiansen P, Balslev E, Kroman N (2015). Prognostic significance of axillary dissection in breast cancer patients with micrometastases or isolated tumor cells in sentinel nodes: a nationwide study.

[23] Sola M, Alberro JA, Fraile M (2013). Complete axillary lymph node dissection versus clinical follow-up in breast cancer patients with sentinel node micrometastasis: final results from the multicenter clinical trial AATRM 048/13/2000.

[24] Grabau D, Dihge L, Ferno M, Ingvar C, Ryden L (2013). Completion axillary dissection can safely be omitted in screen detected breast cancer patients with micrometastases. A decade's experience from a single institution.

[25] Pernas S, Gil M, Benítez A (2009). Avoiding axillary treatment in sentinel lymph node micrometastases of breast cancer: a prospective analysis of axillary or distant recurrence.

[26] Tvedskov TF, Jensen MB, Lisse IM, Ejlertsen B, Balslev E, Kroman N (2012). High risk of non-sentinel node metastases in a group of breast cancer patients with micrometastases in the sentinel node.

[27] Collins M, O'donoghue C, Sun W (2017). Use of axillary lymph node dissection (ALND) in patients with micrometastatic breast cancer.

[28] Galimberti V, Botteri E, Chifu C (2012). Can we avoid axillary dissection in the micrometastatic sentinel node in breast cancer?.

[29] Youssef MM, Cameron D, Pucher PH, Olsen S, Ferguson D (2016). The significance of sentinel lymph node micrometastasis in breast cancer: comparing outcomes with and without axillary clearance.

[30] Houvenaeghel G, Boher JM, Reyal F (2016). Impact of completion axillary lymph node dissection in patients with breast cancer and isolated tumour cells or micrometastases in sentinel nodes.

[31] Pepels MJ, De Boer M, Bult P (2012). Regional recurrence in breast cancer patients with sentinel node micrometastases and isolated tumor cells.

[32] Azghadi S, Daly M, Mayadev J (2016). Practice patterns of radiation field design for sentinel lymph node-positive early-stage breast cancer.

[33] Mamtani A, Patil S, Van Zee KJ (2016). Age and receptor status do not indicate the need for axillary dissection in patients with sentinel lymph node metastases.

[34] De Boer M, Van Deurzen CHM, Van Dijck JaaM (2009). Micrometastases or isolated tumor cells and the outcome of breast cancer.

[35] Onishi T, Jinno H, Takahashi M (2010). Non-sentinel lymph node status and prognosis of breast cancer patients with micrometastatic sentinel lymph nodes.

[36] Bargehr J, Edlinger M, Hubalek M, Marth C, Reitsamer R (2013). Axillary lymph node status in early-stage breast cancer patients with sentinel node micrometastases (0.2–2 mm).

[37] Mamtani A, Patil S, Stempel M, Morrow M (2017). Axillary micrometastases and isolated tumor cells are not an indication for post-mastectomy radiotherapy in stage 1 and 2 breast cancer.

[38] Kumar S, Bramlage M, Jacks LM (2010). Minimal disease in the sentinel lymph node: how to best measure sentinel node micrometastases to predict risk of additional non-sentinel lymph node disease.

[39] Truong PT, Vinh-Hung V, Cserni G (2008). The number of positive nodes and the ratio of positive to excised nodes are significant predictors of survival in women with micrometastatic node-positive breast cancer.

[40] Dillon MF, Hayes BD, Quinn CM (2010). The extent of axillary lymph node clearance required following detection of sentinel node micrometastases.

